# A systematic review protocol investigating tests for physical or physiological qualities and game-specific skills commonly used in rugby and related sports and their psychometric properties

**DOI:** 10.1186/s13643-016-0298-1

**Published:** 2016-07-27

**Authors:** Matthew Chiwaridzo, Gillian D. Ferguson, Bouwien C. M. Smits-Engelsman

**Affiliations:** 1 University of Zimbabwe, College of Health Sciences, Rehabilitation Department, P.O. Box A178, Avondale, Harare Zimbabwe; 2 Division of Physiotherapy, School of Health and Rehabilitation Sciences, Faculty of Health Sciences, University of Cape Town, Observatory Campus, Cape Town, South Africa

**Keywords:** Physical, Physiological characteristics, Rugby, Game-specific skills, Systematic review protocol, Psychometric properties

## Abstract

**Background:**

Scientific focus on rugby has increased over the recent years, providing evidence of the physical or physiological characteristics and game-specific skills needed in the sport. Identification of tests commonly used to measure these characteristics is important for the development of test batteries, which in turn may be used for talent identification and injury prevention programmes. Although there are a number of tests available in the literature to measure physical or physiological variables and game-specific skills, there is limited information available on the psychometric properties of the tests. Therefore, the purpose of this study is to systematically review the literature for tests commonly used in rugby to measure physical or physiological characteristics and rugby-specific skills, documenting evidence of reliability and validity of the identified tests.

**Methods/design:**

A systematic review will be conducted. Electronic databases such as Scopus, MEDLINE via EBSCOhost and PubMed, Academic Search Premier, CINAHL and Africa-Wide Information via EBSCOhost will be searched for original research articles published in English from January 1, 1995, to December 31, 2015, using a pre-defined search strategy. The principal investigator will select potentially relevant articles from titles and abstracts. To minimise bias, full text of titles and abstracts deemed potentially relevant will be retrieved and reviewed by two independent reviewers based on the inclusion criteria. Data extraction will be conducted by the principal investigator and verified by two independent reviewers. The Consensus-based Standards for the Selection of Health Measurement Instruments (COSMIN) checklist will be used to assess the methodological quality of the selected studies.

**Discussion:**

Choosing an appropriate test to be included in the screening test battery should be based on sound psychometric properties of the test available. This systematic review will provide an overview of the tests commonly used in rugby union and other related high intermittent team sports characterised by skill executions using the hands and legs such as Rugby League and Australian Rules Football. In addition, the review will highlight the psychometric properties of the identified tests. This information is crucial in developing a sport-specific test battery which can be used for talent identification, especially among young adolescent players, and injury prevention programmes.

**Systematic review registration:**

PROSPERO CRD42015029747

**Electronic supplementary material:**

The online version of this article (doi:10.1186/s13643-016-0298-1) contains supplementary material, which is available to authorized users.

## Background

Rugby union (henceforth referred to as rugby) is a popular sport played in many countries worldwide at both junior and senior levels [1]. It is characterised by multiple high-intensity activities interrupted shortly by low-intensity activities [2–5]. The players engage in physically demanding contests such as tackles, rucks and mauls in order to gain possession of the ball [6]. This requires players to possess a wide range of physical attributes allowing them to be fatigue-resistant and stronger in physical contests [7, 8]. Today, the physiological and skills profiling of players has become an important aspect of the game to determine competent players ready to meet the high-intensity demands of the sport [7].

Although there are position-specific requirements in rugby, recent evidence point towards a blend of roles since all rugby players are expected to compete and maintain possession of the ball [6, 9, 10]. Consequently, success in professional rugby circles and other related intermittent team sports such as Rugby League and Australian Rules Football (AFL) that involve similar demands has been attributed to exhibiting (but not limited to) well-developed physiological characteristics such as endurance, muscular strength, power, agility, speed and flexibility [11–13]. In addition, high levels of game-specific skills such as tackling, kicking, passing, catching and reactive agility have also been indicated to be very important in the sport [2, 10, 14–17]. There is burgeoning research showing that physiological characteristics and game-specific skills discriminate significantly between players of different ranks and abilities [18, 19]. Understanding the physiological qualities and game-specific skills that discriminate between players in rugby will allow coaches and researchers to prepare highly effective training programmes and develop specific tests to examine players’ proficiencies [20].

There are many different tests available in the literature to measure physical or physiological variables and game-specific skills in rugby (Table 1). At the top level, rugby players are required to perform high-speed running or sprinting during defending or attacking [2, 3]. Speed enables players to move quickly in order to position themselves in attack and defence [21, 22]. According to time-motion studies, rugby players rarely sprint distances greater than 40 m in a single bout of intense activity [21]. Therefore, speed is commonly measured using the sprinting tests performed between distances of 5 and 50 m, whereas speed endurance is usually assessed using repetitive sprinting tests [8, 23–25].

**Table 1:** A summary of selected physiological qualities and game-specific skills needed in rugby and the corresponding test(s)

Construct	Test(s)
1. Physical/motor or physiological qualities	
a. Muscular strength and power	
• Upper body muscular strength	3 repetition maximum bench press [19]; 2-kg medicine ball throw [58]
• Upper body strength endurance	Bench press with repetitions [19]; flexed-arm hang test [36]
• Lower body explosive power	Vertical jump test [19]; standing broad jump [44]; countermovement jump test [26]
• Lower body muscular strength	3 repetition maximum full-body squat [19]
• Abdominal strength	Sit-up test [59]
b. Speed/acceleration	10- and 30-m running test [28]; 10- and 40-m sprint test [23]; 20-m sprint test [18]
c. Agility	Illinois test [34]; *t* test [44]; change of direction speed [26]; zigzag run test [26, 36]
• Reactive agility	Reactive agility test [27]
d. Flexibility	Sit and reach test [5]; adapted sit and reach [36]
e. Aerobic capacity	Yo-Yo intermittent recovery test [19]; 20-m multistage endurance run [59]; multistage fitness test [23]
• Speed endurance	Repetitive sprint test [26]; repeated 20-m sprint test [23]; repeated 12-s sprint shuttle test [23]; speed endurance test (test of Halzadine and McNab) [36]
2. Rugby-specific skills	
a. Ground skills ability	Pick up and place test [34]
b. Passing	4-m passing for accuracy test [59]; 7-m passing for distance ability test [28]
c. Kicking	Kicking ability test [28]; place kicking test; air and ground kicking ability test [36]
d. Catching	Catching ability test while moving [28]; catching and throwing over the crossbar [60, 61]
e. Tackling	One-on-one tackling drill in a 10-m grid proficiency assessed using standardised technical criteria [23]
f. Draw and pass	Single- and dual-task draw and pass assessment test [23]
g. Pattern recall and prediction	Pattern recall and prediction test [23]

Agility is another physical characteristic essential in rugby [26, 27]. Players are required to make fast decisions while rapidly accelerating, decelerating and changing direction [21, 22]. Several authors have evaluated the agility of rugby players using a number of different tests including the ‘L’ run, Illinois agility run test, 505 and the modified 505 test [28, 29]. The pre-planned nature of these tests limit their applicability to real game demands since changes of direction in rugby are often in response to stimuli such as an attacking or defending opponent [25, 27, 30]. It is now commonly accepted that perceptual or neuropsychological factors such as anticipation, intuition, sensory processing and decision-making are all important to agility performance [27, 30]. Today, the reactive agility test (RAT) is widely used in literature to evaluate the change in direction with speed while the players are responding to unpredictable stimuli [27, 30, 31].

Muscular strength and power are also important for success in rugby [3]. Muscular strength has been consistently measured using the back squat for the lower body and the bench press for the upper body, testing either 1 or 3 repetition maximum (RM) [19, 32–34]. Rugby players are required to have high levels of muscular power in order to effectively perform the lifting, pushing and pulling tasks that occur during a match [21, 22]. In addition, muscular power is required for line-out jumping, breaking through tackles and agility when attacking [21, 35]. Muscular power is commonly assessed in the lower body using the vertical jump height test [19, 23, 36].

Rugby players use both aerobic and anaerobic energy systems [37]. Rugby matches last 60–80 min with players covering between 5500 and 9929 m depending on the level of competition, pace of the game and players positions [3, 10, 21]. Given the duration of a rugby match, well-developed aerobic power is important for performance [3]. Numerous studies have measured the aerobic capacity of rugby players by estimating maximal oxygen uptake (VO_2max_) through the multistage fitness test [21, 38–40]. In addition, there are instances where players are required to perform large amounts of high-speed running in a short period of time. As such, well-developed high-intensity running ability is required in order to compete during these periods. Tests commonly used to assess this include the repeated 12-s sprint shuttle test and Yo-Yo Intermittent recovery test level 1 [19, 23, 32, 41].

Rugby-specific skills are also vital for successful performance, and they have been used to differentiate between elite and non-elite players [15, 42]. The basic skills of passing, kicking, running and catching have been reported to represent the fundamental game skills in rugby that are performed by all players [15, 34]. The tests commonly used for passing include passing for accuracy and distance tests, and the tests for kicking include kicking for distance test and place kicking test using a tee, air and ground kicking ability test [28, 34, 36]. Side-stepping ability is usually tested using the side-step ability test involving carrying the ball in both hands running through obstacles and sidestepping to the left and right [36]. Ground skills test is used to assess ground skills ability while running and involves the player picking up the ball in both hands and running around the marker and placing the ball where it was picked while running [36]. Arguably, tackling is one of the core skills needed in rugby by all players. In several studies, tackling proficiency was assessed subjectively by expert rugby coaches based on a standardised skill criteria rating the skill on a Likert scale ranging from 1 (lowest score) to 5 (optimal score) with the players either playing game-related training activities or competing in competitive matches [15, 18, 23].

Identification of tests commonly used to measure physiological or physical qualities and rugby-specific skills is important for the development of screening test batteries. Test batteries can be used by coaches and researchers to determine players’ competency level, especially for the purpose of talent identification, creating a profile of each individual athlete, tracking progress over time and also evaluating the effectiveness of interventions [43–46]. However, choosing an appropriate test to use for practical or research purposes should be based on the test displaying acceptable psychometric properties [47, 48]. This is extremely important in sports science as in medical and health-related fields to know which tests are indeed reliable and valid. To help rugby sport coaches to determine appropriate tests to include in the evaluation of physical characteristics and rugby skills, the evidence for reliability and validity of the tests should be considered [49]. Despite the widespread use of physical or game-specific skills in the literature, studies exploring the psychometric properties of tests commonly used in rugby are limited. To the authors’ knowledge, no systematic review has been conducted to review the psychometric properties of the tests commonly used to measure physiological characteristics and game-specific skills in rugby. A recent systematic review conducted by Robertson et al. [47] reviewed 22 studies to determine the psychometric properties of tests for skills from a broad range of sports. Only one study investigating rugby league was included in the review. However, the majority (95 %) of the reviewed studies investigated test-retest reliability (95 %) and content validity (68 %).

### Objectives

The purpose of this systematic review is twofold and, hence, will be conducted in two stages.

Stage 1

1. The specific objective is to determine the range of tests, used alone or included in test batteries, commonly used to measure physical or physiological characteristics and game-specific skills needed in rugby union and other high intermittent team sports characterised by skill executions using the hands and legs such as Rugby League and Australian Rules Football (AFL).

Stage 2

1. The specific objective of stage 2 is to document the psychometric properties of the previously identified tests.

## Methods

### Study design

A systematic review will be conducted. This systematic review forms part of a broader doctoral study with the ultimate aim of developing a screening test battery encompassing validated tests. Once developed, the scores for the test battery will be used to predict the risk of injury in a prospective study among Zimbabwe male adolescent elite rugby players. In Zimbabwe, rugby is a popular sport played competitively by males [50] and this accounts for the specific focus on males. The full doctoral thesis is planned around three phases. In the first phase, a narrative literature review will be conducted first to describe the qualities and skills needed in the game of rugby and identify the commonly used tests for the identified variables. Subsequently, a small-scale qualitative study using interviews will be conducted to explore the perceptions of local rugby coaches on the qualities and skills crucial in rugby and the tests they administer to evaluate the identified factors. This systematic review will form the last part of the first phase and will then be conducted mainly to report on the evidence of the psychometric properties of commonly used tests for game-specific skills and physical or physiological variables. This review will largely be informed by conventional methods of conducting systematic reviews and will be written in accordance with the Preferred Reporting Items for Systematic Review and Meta-Analysis Protocol (PRISMA-P) guidelines by Moher et al. [51] (see Additional file 1).

### Study registration

The protocol has been registered on PROSPERO with the registration number CRD42015029747.

### Criteria for inclusion of studies

#### Study design

One of the main principles of a systematic review is to include all available evidence and then summarise narratively or quantitively the findings [52]. Therefore, there will be no restrictions on the type of study to be included in the review.

#### Sport context

Although rugby union differs subtly from rugby league in rules, scoring and patterns of play, the similarities are not only in game duration, field size, player positions and goal posts but also in the physical demands, physiological responses of players during play and the technical or perceptual skills needed in the sports [53]. Therefore, this systematic review will include articles on all rugby codes (i.e. rugby league) and other related sports such as Rugby League and AFL with similar executions of skills by both hands and legs characterised by multiple high-intensity activities (e.g. high-speed running and sprinting) interrupted intermittently by low-intensity activities.

#### Outcome measures

Rugby players have to exhibit a blend of physical or physiological and rugby-specific skills to cope with the demands of the game [28]. To be included in this review, studies should report on the following two concepts: (a) physical or physiological characteristics and (b) rugby-specific skills. In addition, studies should provide detailed information on the procedure used to measure any of the aforementioned qualities and the instrument or test used in measuring in their methods section. Explicitly expressed in the text of the studies to be included in this review should be information at least on one psychometric property used to evaluate the test/instrument and the results obtained for the measurement property.

#### Participants

Rugby is played competitively and professionally from secondary school to senior club level worldwide [28, 54]. Studies to be included in this systematic review should have detailed information about the participants. Studies reported for adolescents considered from above 10 years through to adults will be considered. In this review, studies involving male adults or adolescent rugby union or league players will be included since the identified tests will be used in males who participate in competitive rugby.

To answer the second objective, all the studies included in stage 1 will be evaluated for psychometric properties of the test(s) included in the study. The studies will be included if they state explicitly information on at least one psychometric property tested for the included test(s), even if the primary objective of the study is not on psychometric properties. Articles utilising tests with measurement properties investigated previously elsewhere, provided that the validation involved rugby participants, will also be included since the purpose of this review is to document the evidence of reliability and validity of the test. In addition, articles primarily evaluating the psychometric properties of a test identified in the previous stage as commonly used will also be included provided that the test is designed for and tested among rugby or rugby-related sports participants. However, to be able to assess the methodological quality of these studies, they have to provide detailed information on the procedure of the included test(s) for the mentioned quality or skill.

### Exclusion criteria

Studies that do not fulfil any of the inclusion criteria will be excluded. Non-scholarly documents will be excluded; these include thesis, editorials, newspaper articles and lecture notes as suggested in the literature [48]. In addition, studies not published in English will be excluded as the authors are predominantly from English-speaking countries and the review has no funding to fund the back and forth translation of non-English articles. No restriction criteria will be applied for country. Articles describing tests for physical or physiological qualities and game-specific skills on rugby participants living with disabilities such as quadriplegics will be excluded as the technical and physiological skills needed could be different.

### Search methods for identification of studies

A computerised, systematic literature search will be conducted in electronic databases such as Scopus, MEDLINE via EBSCOhost and PubMed, Academic Search Premier, CINAHL (Cumulative Index to Nursing and Allied Health Literature) and Africa-Wide Information via EBSCOhost. In accordance with recommendations for systematic reviews on measurement properties [55], a hand search will be done on the reference lists of included articles to identify additional relevant studies. In addition, the Science Citation Index for citation searching will also be used to search for articles. There has been an exponential increase in the volume of scientific research on rugby after the sport attained professional status in 1995 [6]. Hence, this review will include articles published in the last 20 years between January 1, 1995, and December 31, 2015.

### Search strategy

Studies on psychometric properties of measurement instruments have been reported to be difficult to find in literature especially on PubMed [48]. This has been attributed to indexing problems, large variation in terminology used for measurement properties and the poor reporting of measurement properties in abstracts [48]. Therefore, a search strategy proposed by Terwee et al. [48] will be used as a guide to the selection of the key or index terms to be used when searching for articles. The search strategy was developed in consultation with an expert librarian (GM) in systematic review from the University of Cape Town. Additional file 2 shows the search strategy to be used in PubMed and will consist of a combination of the following search themes connected with the Boolean term AND:

i. Construct-related search terms, for example, physical OR physiological OR rugby skill*
ii. Population-related search terms, for example, adult OR senior OR adolescen* OR youth
iii. Sport-related search terms, for example, rugby OR rugby union OR rugby league
  The above search strategy will constitute stage 1 and will be used to provide an overview of the tests commonly used in the literature to measure physiological variables and game-specific skills. The search strategy below will be used in stage 2 to determine the psychometric properties of the identified tests. Including the ‘sport-related’ search terms used in stage 1, the search strategy for stage 2 will additionally include ‘instrument-related search terms’ and ‘measurement properties-related search terms’. The search strategy consisted of a combination of the following search themes connected with the Boolean terms AND:
iv. Instrument-related terms, for example, vertical jump test* OR multistage fitness test* OR repeated ability sprint test*
v. Measurement properties-related terms, for example, psychometr* OR clinimetr* OR clinometr* OR reproducib* OR reliab*

For ‘instrument-related’ search terms, the specific names of the tests identified in the first stage of the review will be entered to search for their psychometric properties.

### Selection process

The selection process will then be conducted as recommended by van Tulder et al. [52] and Reimers et al. [55]. The principal investigator will apply the inclusion criteria to select potentially relevant articles from titles and abstracts. Before the screening phase, all the search results will be merged in Rev Manager Version 5.3 to identify and remove duplicates. The full text of titles and abstracts deemed potentially relevant will be retrieved, and two independent reviewers (GF and EB) will review the full-text articles for inclusion using pre-defined eligibility criteria. Any disagreements that arise will be resolved through discussion or referral to a third party (BE).

### Data management

Eligible articles gathered for the systematic review will be downloaded and stored in a Dropbox folder accessible to all the authors. An account will be created by the principal investigator (MC) in the respective databases used to retrieve articles. Hence, the online version of the articles and the electronic search strategy used for each database will be saved therein.

### Data extraction

After identification and complete analysis of the full-text articles for eligibility, the primary author will extract data from each article into a Microsoft Excel data collection form. For the first objective, the following data will be captured: the publication details (first author, year of publication), the name(s) of the physiological or physical characteristic or game-specific skill examined in the study and the corresponding test(s) used to measure the variables. The frequency of use of each test in all the included studies will also be reported. To be able to describe the characteristics of the studies included in the review, additional information on sport context, age of participants, country and target population will also be extracted. Thereafter, the extracted data will be assessed by two reviewers (SO and JMD) for accuracy against the original sources.

For the second objective on psychometric properties, the following data will also be captured: the publication details (first author, title, year of publication), study and subject characteristics, sport context, name of the test (s) used, a short description of its procedure, psychometric properties reported (reliability, internal consistency, measurement error/smallest detectable difference, content validity, construct validity, responsiveness) and the results obtained. Two independent assessors (JMD and SO) will verify the extracted data for accuracy and consistency against the original articles.

### Outcomes and prioritisation

For this review, the primary outcome measures are tests used in rugby and related sports commonly to measure physical or physiological characteristics and rugby-specific skills. Secondarily, evidence of measurement properties such as reliability and validity will be captured for each identified test for the qualities and skills needed in the sport of rugby.

### Methodological quality assessment

Methodological quality assessment of included studies is a necessary part of systematic review [56]. In order to assess the overall quality of the selected articles, the ‘Consensus-based Standards for the Selection of Health Measurement Instruments’ (COSMIN) checklist will be used as a guide. The checklist is a standardised tool for evaluating the rigour of psychometric studies of measurement instruments, and only the methodological part of the checklist will be used [54, 55]. It evaluates nine psychometric properties of internal consistency, reliability, measurement error, content validity, construct validity (i.e. structural validity, hypothesis testing, and cross-cultural validity), criterion validity, responsiveness, interpretability and generalisability (Table 2) [56, 57].

**Table 2:** Quality of the statistical outcomes to determine psychometric properties [57, 62]

Measurement property	Definition	(Rating) quality criteria^a,b^
Reliability		
Internal consistency	The extent to which items in a (sub)scale are intercorrelated, thus measuring the same construct	(+) Factor analyses performed on adequate sample size (7 * # items and >100) AND Cronbach’s alpha(s) calculated per dimension AND Cronbach’s alpha(s) between 0.70 and 0.95 (?) No factor analysis OR doubtful design or method (−) Cronbach’s alpha(s) 0.70 or O 0.95, despite adequate design and method (0) No information found on internal consistency
Reproducibility		
Agreement	The extent to which the scores on repeated measures are close to each other (absolute measurement error)	(+) MIC < SDC OR MIC outside the LOA OR convincing arguments that agreement is acceptable (?) Doubtful design or method OR (MIC not defined AND no convincing arguments that agreement is acceptable) (−) MIC > SDC OR MIC equals or inside LOA, despite adequate design and method; (0) No information found on agreement
Reliability	The extent to which patients can be distinguished from each other, despite measurement errors (relative measurement error)	(+) ICC > 0.70 OR *k* > 0.70 (?) Doubtful design or method (e.g. time interval not mentioned) (−) ICC or weighted Kappa ≤0.70, despite adequate design and method (0) No information on reliability found
Validity		
Content validity	The extent to which the domain of interest is comprehensively sampled by the items in the questionnaire	(+) A clear description is provided of the measurement aim, the target population, the concepts that are being measured, and the item selection AND target population and (investigators OR experts) were involved in the item selection (?) A clear description of the above-mentioned aspects is lacking OR only target population involved OR doubtful design or method (−) No target population involvement (0) No information found on target population involvement
Construct validity	The extent to which scores on a particular questionnaire relate to other measures in a manner that is consistent with theoretically derived hypotheses concerning the concepts that are being measured	(+) Specific hypotheses were formulated AND at least 75 % of the results are in accordance with these hypotheses (?) Doubtful design or method (e.g. no hypotheses) (−) Less than 75 % of hypotheses were confirmed, despite adequate design and methods (0) No information found on construct validity
Criterion validity (predictive or concurrent	The extent to which scores on a particular questionnaire relate to a gold standard	(+) Correlation with standard ≥0.70 OR no statistically significant differences between the two tests found OR sensitivity and specificity ≥0.70 OR convincing arguments that gold standard is ‘gold’ AND correlation with gold standard >0.70^c^ (?) No convincing arguments that gold standard is ‘gold’ OR doubtful design or method (−) Correlation with standard <0.70 or AUC < 0.70 OR statistically significant differences between outcome measures and gold standard OR sensitivity or specificity <0.70
Responsiveness	The ability of a questionnaire to detect clinically important changes over time	(+) SDC or SDC < MIC OR MIC outside the LOA OR RR O 1.96 OR AUC > 0.70 (?) Doubtful design or method (−) SDC or SDC > MIC OR MIC equals or inside LOA OR RR < 1.96 OR AUC < 0.70, despite adequate design and methods (0) No information found on responsiveness
Floor and ceiling effects	The number of respondents who achieved the lowest or highest possible score	(+) ≤15 % of the respondents achieved the highest or lowest possible score (?) Doubtful design or method (−) >15 % achieved the highest and lowest possible score despite adequate designs and methods (0) No information found on interpretation
Interpretability	The degree to which one can assign qualitative meaning to quantitative scores	(+) Mean and SD scores presented of at least 4 relevant subgroups of patients and MIC defined (?) Doubtful design or method OR less than 4 subgroups OR no MIC defined (0) No information found on interpretation

*MIC* minimal important change, *SDC* smallest detectable change, *LOA* limits of agreement, *ICC* intraclass correlation, *SD* standard deviation

^a^(+) positive rating; (?) indeterminate rating; (−) negative rating; (0) no information available

^b^Doubtful design or method = lack of a clear description of the design or methods of the study, sample size smaller than 50 subjects (should be at least 50 in every (subgroup) analysis), or any important methodological weakness in the design or execution of the study

^c^Adopted from van Bloemendaal et al. [26]

Two authors (EB and GF) will be used to assess the quality and calculate the score of the included articles. Disagreements between reviewers will be resolved by discussion or the use of a third person (BE). The scoring system is designed that items are scored as ‘excellent’ when there is evidence of adequate methodological quality, ‘good’ when relevant information is not fully reported but adequate quality can be assumed, ‘fair’ if the methodological quality is in doubt and ‘poor’ when there is evidence that the methodological quality is not adequate [55]. Each psychometric property will be evaluated separately by taking the lowest rating of any item based on the 4-point scale from excellent, good, fair and poor [56].

### Quality criteria for measurement properties

The Quality Criteria for Measurement Properties (Table 2) as given by Terwee et al. [57] will be used to rate each psychometric property in the articles as positive, negative or questionable depending on the results of the property reported. Test-retest reliability or interrater reliability will be considered substantial for intraclass correlation coefficients (ICC) above 0.70 [57]. In addition, tests will be considered to have acceptable construct validity if the effect size (ES) between groups is as follows: <0.2 trivial, 0.2–0.5 small, 0.5–0.8 medium and >0.8 large [2]. In the case of the effect size not reported in any of the included articles, the authors of the respective studies will be contacted directly for a maximum of three times through email to either provide the information necessary for its calculation or provide the actual effect size. If no response is received from the authors after the third attempt, provided all the parameters are available, the effect size will be calculated by the primary investigator and will be reported as ‘calculated’.

### Data synthesis

A narrative synthesis of the findings from the included studies will be provided due to the likely heterogeneity of the studies. A pilot testing of the search strategy for both stages of this review showed a number of different studies. In that case, a narrative synthesis may be necessary to provide potential explanations for contrasting findings observed in the literature, summarising the information in tables and explaining in text the characteristics and findings of the included studies for both stages of the review.

### Risk of bias in individual studies

The COSMIN checklist will be used for assessing the methodological quality of all the studies to be included in the review. The use of the COSMIN to this effect precludes the possibility of selecting and evaluating individual studies reporting on tests that were developed using designs with poor methodological rigour.

## Discussion

The purpose of this systematic review is to identify tests for physical or physiological and game-specific skills that are psychometrically sound and that can be amalgamated in a test battery for use in rugby. Identification of tests commonly used to measure these characteristics is important for the development of multidimensional test batteries integrating all essential qualities needed in rugby. The test batteries will enable the recognition and development of talented rugby players at an early age.

## Abbreviations

AFL, Australian Rules Football; AUC, area under the curve; CINAHL, Cumulative Index to Nursing and Allied Health Literature; COSMIN, Consensus-based Standards for the Selection of Health Measurement Instruments; ES, effect size; ICC, intraclass correlation coefficient; LOA, limits of agreement; MIC, minimal important change; PRISMA-P, Preferred Reporting Items for Systematic Review and Meta-Analysis Protocol; SD, standard deviation; SDC, smallest detectable change

## Additional files

Additional file 1: PRISMA-P guidelines for systematic review protocols. (DOCX 13.8 kb) Additional file 2: Search strategy. (DOCX 13.9 kb)
