# Supplementary material for: A systematic review protocol investigating tests for physical or physiological qualities and game-specific skills commonly used in rugby and related sports and their psychometric properties
Source: Syst Rev. 2016 Jul 27;5:122. doi: 10.1186/s13643-016-0298-1 (PMC4962394; doi:10.1186/s13643-016-0298-1)
Supplement: Additional file 1: — PRISMA-P guidelines for systematic review protocols. (DOCX 13.8 kb) [file 13643_2016_298_MOESM1_ESM.docx]

**Additional file 1: Prisma-P guidelines applicable**

| **Item** | **Description** | **Checklist** |
| --- | --- | --- |
| **Title** |  |  |
| Identification | Identify the report as a protocol of a systematic review | **√** |
| **Registration** | If registered, provide the name of the registry (e.g., PROSPERO) and registration number | **Registered** |
| **Authors** |  |  |
| Contact | Provide name, institutional affiliation, and e-mail address of all protocol authors; provide physical mailing address of corresponding author | **√** |
| Contributions | Describe contributions of protocol authors and identify the guarantor of the review | **√** |
| **Support** |  |  |
| Sources | Indicate sources of financial or other support for the review | **√** |
| Sponsor | Provide name for the review funder and/or sponsor | **√** |
| Role of sponsor/funder | Describe roles of funder(s), sponsor(s), and/or institution(s), if any, in developing the protocol | **√** |
| **Introduction** |  |  |
| Rationale | Describe the rationale for the review in the context of what is already known | **√** |
| Objectives | Provide an explicit statement of the question(s) the review will address with reference to participants, interventions, comparators, and outcomes (PICO) | **√** |
| **Methods** |  |  |
| Eligibility criteria | Specify the study characteristics (e.g., PICO, study design, setting, time frame) and report characteristics (e.g., years considered, language, publication status) to be used as criteria for eligibility for the review | **√** |
| Information sources | Describe all intended information sources (e.g., electronic databases, contact with study authors, trial registers, or other grey literature sources) with planned dates of coverage | **√** |
| Search strategy | Present draft of search strategy to be used for at least one electronic database, including planned limits, such that it could be repeated | **√** |
| **Study records** |  |  |
| **Data management** | Describe the mechanism(s) that will be used to manage records and data throughout the review | **√** |
| **Selection process** | State the process that will be used for selecting studies (e.g., two independent reviewers) through each phase of the review (i.e., screening, eligibility, and inclusion in meta-analysis | **√** |
| **Data collection process** | Describe planned method of extracting data from reports (e.g., piloting forms, done independently, in duplicate), any processes for obtaining and confirming data from investigators | **√** |
| **Data items** | List and define all variables for which data will be sought (e.g., PICO items, funding sources), any pre-planned data assumptions and simplifications | **√** |
| **Outcomes and prioritisation** | List and define all outcomes for which data will be sought, including prioritization of main and additional outcomes, with rationale | **√** |
| **Risk of bias in individual studies** | Describe anticipated methods for assessing risk of bias of individual studies, including whether this will be done at the outcome or study level, or both; state how this information will be used in data synthesis | **√** |
