# Supplementary material for: A systematic review protocol investigating tests for physical or physiological qualities and game-specific skills commonly used in rugby and related sports and their psychometric properties
Source: Syst Rev. 2016 Jul 27;5:122. doi: 10.1186/s13643-016-0298-1 (PMC4962394; doi:10.1186/s13643-016-0298-1)
Supplement: Additional file 2: — Search strategy. (DOCX 13.9 kb) [file 13643_2016_298_MOESM2_ESM.docx]

**Additional file 2: Search strategy**

**Stage 1: PubMed Strategy**

Physical OR physical skill* OR physical characteristic* OR physical fitness OR motor skill* motor abilit* OR motor component* OR movement characteristic* OR performance analys* OR performance OR physiological OR physiological characteristic* OR physiological variable* OR technical skill* OR perceptual skill* OR rugby-specific skill* game specific skill* OR skill* OR rugby skill* OR skill testing* OR expertise OR skill attribute

Adult OR senior OR adolescent* OR youth OR teenager* OR elite OR sub-elite OR male* OR U-16 players OR U-18 players OR professional OR amateur OR boy*OR junior

Rugby OR rugby union OR rugby union team OR rugby league OR rugby player* OR elite rugby players OR team sport* OR collision sport* OR talented OR talent identification OR talent selection OR non-talented OR draft* or non-draft* OR skilled players OR positional differences OR Australian Rules football OR Australian Football OR American football

Test* OR screening test OR screening OR testing battery* OR field test OR physical test* OR physical performance test OR instrument* OR outcome measure* OR measure* OR tool* OR screening tool*

**Stage 2: PubMed Strategy**

Psychometrics OR psychometr* OR psychometric property* OR clinimetr* OR clinometr* OR clinimetric property* OR measurement property OR reproducibility of results OR reproducib* OR reliab* OR test-retest OR intra-rater OR inter-rater OR measurement error OR valid* OR construct valid* OR face valid*OR validation OR discriminative validity OR concurrent valid* OR convergent valid*

Vertical jump test* OR speed test* OR repeated 20m sprint test* OR multistage fitness stage test* OR L-run test* OR Yo-yo intermittent recovery (level 1) test* OR 1RM bench press test*

*[(((((physical OR physical skill* OR physical characteristic* OR physical fitness OR motor skill* motor abilit* OR motor component* OR movement characteristic* OR performance analys* OR performance OR physiological OR physiological characteristic* OR physiological variable* OR technical skill* OR perceptual skill* OR rugby-specific skill* game specific skill* OR skill* OR rugby skill* OR skill testing* OR expertise OR skill](http://www-ncbi-nlm-nih-gov.ezproxy.uct.ac.za/pubmed?term=%28%28%28%28%28physical%20OR%20physical%20skill*%20OR%20physical%20characteristic*%20OR%20physical%20fitness%20OR%20motor%20skill*%20motor%20abilit*%20OR%20motor%20component*%20OR%20movement%20characteristic*%20OR%20performance%20analys*%20OR%20performance%20OR%20physiological%20OR%20physiological%20characteristic*%20OR%20physiological%20variable*%20OR%20technical%20skill*%20OR%20perceptual%20skill*%20OR%20rugby-specific%20skill*%20game%20specific%20skill*%20OR%20skill*%20OR%20rugby%20skill*%20OR%20skill%20testing*%20OR%20expertise%20OR%20skill%20attributes%29%29%29%20AND%20%28%28adult%20OR%20senior%20OR%20adolescent*%20OR%20youth%20OR%20teenager*%20OR%20elite%20OR%20sub-elite%20OR%20male%20OR%20u-16%20players%20OR%20u-18%20players%20OR%20professional%20OR%20amateur%20AND%20rugby%20OR%20rugby%20union%20OR%20rugby%20union%20team%20OR%20rugby%20league%20OR%20rugby%20player*%20OR%20elite%20rugby%20players%20OR%20team%20sport*%20OR%20collision%20sport*%20OR%20talented%20OR%20talent%20identification%20OR%20talent%20selection%20OR%20non-talented%20OR%20draft*%20OR%20non-draft*%20OR%20skilled%20players%20OR%20positional%20differences%29%29%29%20AND%20%28%28Test*%20OR%20screening%20test%20OR%20screening%20OR%20testing%20battery*%20OR%20field%20test%20OR%20physical%20test*%20OR%20physical%20performance%20test%20OR%20instrument*%20OR%20outcome%20measure*%20OR%20measure*%20OR%20tool*%20OR%20screening%20tool*%29%29%29%20AND%20%28%28Psychometrics%5bMeSH%5d%20OR%20psychometr*%20OR%20psychometric%20property*%20OR%20clinimetr*%20OR%20clinometr*%20OR%20clinimetric%20property*%20OR%20measurement%20property%20OR%20reproducibility%20of%20results%5bMeSH%5d%20OR%20reproducib*%20OR%20reliab*%20OR%20test-retest%20OR%20intra-rater%20OR%20inter-rater%20OR%20co-efficient%20OR%20internal%20consistency%20OR%20alpha%20cronbach*%20OR%20measurement%20error%20OR%20valid*%20OR%20construct%20valid*%20OR%20content%20valid*%20OR%20face%20valid*or%20validation%20OR%20discriminative%29%29&cmd=correctspelling)* ***[attributes](http://www-ncbi-nlm-nih-gov.ezproxy.uct.ac.za/pubmed?term=%28%28%28%28%28physical%20OR%20physical%20skill*%20OR%20physical%20characteristic*%20OR%20physical%20fitness%20OR%20motor%20skill*%20motor%20abilit*%20OR%20motor%20component*%20OR%20movement%20characteristic*%20OR%20performance%20analys*%20OR%20performance%20OR%20physiological%20OR%20physiological%20characteristic*%20OR%20physiological%20variable*%20OR%20technical%20skill*%20OR%20perceptual%20skill*%20OR%20rugby-specific%20skill*%20game%20specific%20skill*%20OR%20skill*%20OR%20rugby%20skill*%20OR%20skill%20testing*%20OR%20expertise%20OR%20skill%20attributes%29%29%29%20AND%20%28%28adult%20OR%20senior%20OR%20adolescent*%20OR%20youth%20OR%20teenager*%20OR%20elite%20OR%20sub-elite%20OR%20male%20OR%20u-16%20players%20OR%20u-18%20players%20OR%20professional%20OR%20amateur%20AND%20rugby%20OR%20rugby%20union%20OR%20rugby%20union%20team%20OR%20rugby%20league%20OR%20rugby%20player*%20OR%20elite%20rugby%20players%20OR%20team%20sport*%20OR%20collision%20sport*%20OR%20talented%20OR%20talent%20identification%20OR%20talent%20selection%20OR%20non-talented%20OR%20draft*%20OR%20non-draft*%20OR%20skilled%20players%20OR%20positional%20differences%29%29%29%20AND%20%28%28Test*%20OR%20screening%20test%20OR%20screening%20OR%20testing%20battery*%20OR%20field%20test%20OR%20physical%20test*%20OR%20physical%20performance%20test%20OR%20instrument*%20OR%20outcome%20measure*%20OR%20measure*%20OR%20tool*%20OR%20screening%20tool*%29%29%29%20AND%20%28%28Psychometrics%5bMeSH%5d%20OR%20psychometr*%20OR%20psychometric%20property*%20OR%20clinimetr*%20OR%20clinometr*%20OR%20clinimetric%20property*%20OR%20measurement%20property%20OR%20reproducibility%20of%20results%5bMeSH%5d%20OR%20reproducib*%20OR%20reliab*%20OR%20test-retest%20OR%20intra-rater%20OR%20inter-rater%20OR%20co-efficient%20OR%20internal%20consistency%20OR%20alpha%20cronbach*%20OR%20measurement%20error%20OR%20valid*%20OR%20construct%20valid*%20OR%20content%20valid*%20OR%20face%20valid*or%20validation%20OR%20discriminative%29%29&cmd=correctspelling)****[))) AND ((adult OR senior OR adolescent* OR youth OR teenager* OR elite OR sub-elite OR male OR u-16 players OR u-18 players OR professional OR amateur AND rugby OR rugby union OR rugby union team OR rugby league OR rugby player* OR elite rugby players OR team sport* OR collision sport* OR talented OR talent identification OR talent selection OR non-talented OR draft* OR non-draft* OR skilled players OR positional differences))) AND ((Test* OR screening test OR screening OR testing battery* OR field test OR physical test* OR physical performance test OR instrument* OR outcome measure* OR measure* OR tool* OR screening tool*))) AND ((Psychometrics [MeSH] OR psychometr* OR psychometric property* OR clinimetr* OR clinometr* OR clinimetric property* OR measurement property OR reproducibility of results[MeSH] OR reproducib* OR reliab* OR test-retest OR intra-rater OR inter-rater OR co-efficient OR internal consistency OR alpha cronbach* OR measurement error OR valid* OR construct valid* OR content valid* OR face valid*or validation OR discriminative))](http://www-ncbi-nlm-nih-gov.ezproxy.uct.ac.za/pubmed?term=%28%28%28%28%28physical%20OR%20physical%20skill*%20OR%20physical%20characteristic*%20OR%20physical%20fitness%20OR%20motor%20skill*%20motor%20abilit*%20OR%20motor%20component*%20OR%20movement%20characteristic*%20OR%20performance%20analys*%20OR%20performance%20OR%20physiological%20OR%20physiological%20characteristic*%20OR%20physiological%20variable*%20OR%20technical%20skill*%20OR%20perceptual%20skill*%20OR%20rugby-specific%20skill*%20game%20specific%20skill*%20OR%20skill*%20OR%20rugby%20skill*%20OR%20skill%20testing*%20OR%20expertise%20OR%20skill%20attributes%29%29%29%20AND%20%28%28adult%20OR%20senior%20OR%20adolescent*%20OR%20youth%20OR%20teenager*%20OR%20elite%20OR%20sub-elite%20OR%20male%20OR%20u-16%20players%20OR%20u-18%20players%20OR%20professional%20OR%20amateur%20AND%20rugby%20OR%20rugby%20union%20OR%20rugby%20union%20team%20OR%20rugby%20league%20OR%20rugby%20player*%20OR%20elite%20rugby%20players%20OR%20team%20sport*%20OR%20collision%20sport*%20OR%20talented%20OR%20talent%20identification%20OR%20talent%20selection%20OR%20non-talented%20OR%20draft*%20OR%20non-draft*%20OR%20skilled%20players%20OR%20positional%20differences%29%29%29%20AND%20%28%28Test*%20OR%20screening%20test%20OR%20screening%20OR%20testing%20battery*%20OR%20field%20test%20OR%20physical%20test*%20OR%20physical%20performance%20test%20OR%20instrument*%20OR%20outcome%20measure*%20OR%20measure*%20OR%20tool*%20OR%20screening%20tool*%29%29%29%20AND%20%28%28Psychometrics%5bMeSH%5d%20OR%20psychometr*%20OR%20psychometric%20property*%20OR%20clinimetr*%20OR%20clinometr*%20OR%20clinimetric%20property*%20OR%20measurement%20property%20OR%20reproducibility%20of%20results%5bMeSH%5d%20OR%20reproducib*%20OR%20reliab*%20OR%20test-retest%20OR%20intra-rater%20OR%20inter-rater%20OR%20co-efficient%20OR%20internal%20consistency%20OR%20alpha%20cronbach*%20OR%20measurement%20error%20OR%20valid*%20OR%20construct%20valid*%20OR%20content%20valid*%20OR%20face%20valid*or%20validation%20OR%20discriminative%29%29&cmd=correctspelling)* (208 items)

Scopus results
